# Is maxillary diastema an appropriate site for implantation in rats?

**DOI:** 10.1186/s40729-019-0203-5

**Published:** 2020-02-26

**Authors:** Gang Yue, Husham Edani, Andrew Sullivan, Shuying Jiang, Hamed Kazerani, Mohammad Ali Saghiri

**Affiliations:** 1 Department of Periodontics, Rutgers School of Dental Medicine, Newark, NJ USA; 2 The Office of Institutional Assessment, Rutgers School of Dental Medicine, Newark, NJ USA; 3 Department of Restorative Dentistry, Rutgers School of Dental Medicine, Newark, NJ 07103 USA; 4 Department of Endodontics, University of the Pacific, Arthur A. Dugoni School of Dentistry, San Francisco, CA USA

**Keywords:** Implant, Diastema, Rat, Micro-CT

## Abstract

**Background:**

Implantology or implant dentistry is growing fast during last four decades. Facing the growing demand of implant treatment, there are extreme challenges to clinicians and researchers. First is peri-implantitis with remarkable prevalence. Though investigators have revealed that the etiology of the peri-implant infection is similar to periodontitis, clinically there is no effective treatment. Second, implantation in patients with severe systemic conditions, i.e., severe diabetes, lupus, osteoporosis, organ transplant, and cancer with intensive radiotherapy and/or chemotherapy, is another challenge to implant treatment for lack of scientific research data. Animal models are crucial to help investigators reveal the mechanisms underlying these disorders. Murine models are used most commonly. Rats are the better subject in dental implant research, due to mice could not provide clinical compatible and macro-level measurable data for implant osseointegration and peri-implantitis in oral cavity for lacking enough cancellous bone to support an implant more than 1 mm in length.

**Objective:**

Our aim of this research is to find a clinical comparable rat dental implant model.

**Methods:**

Six male Sprague-Dawley rats with body weight more than 500 g were used in the experiment. Each rat received two implants. One implant was placed at maxillary diastema in each side. Seven weeks after the implantation, only one implant successfully osseointegrated without movement and inflammation. Implant success and failure rate is analyzed by using Clopper-Pearson’s exact method at 95% confidence interval.

**Results:**

The present data indicate that the true success rate of implantation in maxillary natural diastema in rat is less than 38.4% at a confident level of 95%. Meanwhile, Micro-CT indicates maxillary first molar position will be a promising site for implantation.

**Conclusion:**

Maxillary nature diastema may not be an appropriate site for implantation research for its low successful rate, but maxillary first molar position could be a candidate for implantation research. Further researches are required to illustrate the details.

## Highlights

- Current study indicates that maxillary nature diastema could be a site to place implant, but it has a low successful rate. Data indicate that the true success rate of implantation in maxillary natural diastema in rat is less than 38.4% at a confident level of 95%. Therefore, it is not an appropriate site for dental implant experiment. Moreover, it may be able to form certain osseointegration, but it could not provide enough cancellous bone to support an implant and further allow to induction of peri-implantitis on this implant. Further experiments are required to improve the successful rate of implant placement at maxillary nature diastema.
- Upon the intensively monitored body weight change during the experiment, rat body weight did not show abrupt changes after implantation though a slight decrease body weight at 2 g was observed 1 week after implantation, indicating the implantation surgery would not cause large interference in systemic conditions.
  Current investigation revealed a potential site that is maxillary 1st molar socket. After extraction of the maxillary 1st molar, the socket can maximally provide cancellous bone to support an implant 2 mm × 3 mm. Further experiment is needed to illustrate the details.

## Background

**Fig. 1:**
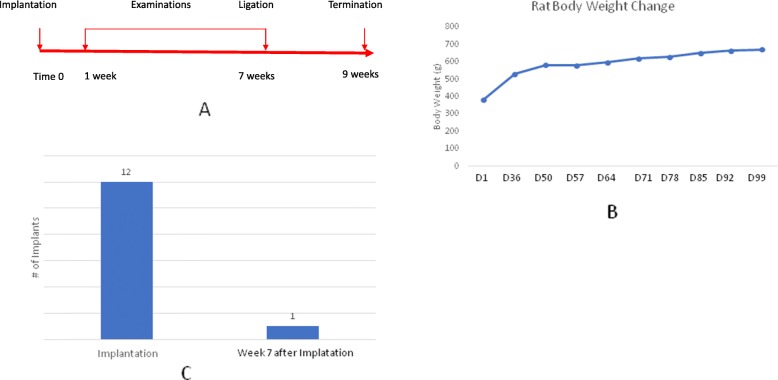
**a**) Time course of the experiment. **b**) Curve of the rat body weight change. **c**) Implant survival after 7 weeks of implantation. Implant success and failure rate is analyzed by using Clopper-Pearson’s exact method at 95% confidence interval. Our experiment data indicate that the true success rate of implantation in maxillary natural diastema in rat is less than 38.4% at a confident level of 95%

**Fig. 2:**
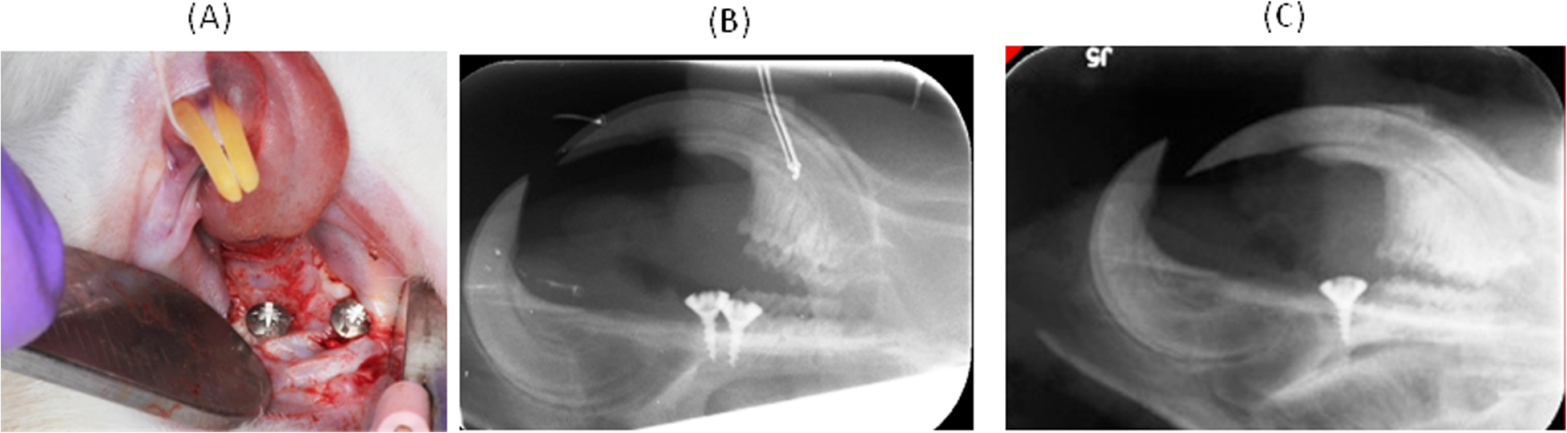
Implantation of two implants at maxillary diastemata per rat. **a** Surgery implantation. **b** X-ray periapical picture after surgery. **c** Seven weeks after implantion

Implantology or implant dentistry is a fast-growing industry. It is reported that the global dental implant market was valued at $ 3.77 billion in 2016 growing at a compound annual growth rate (CAGR) of 7.7% over the forecast period (2018–2024) [1]. The USA holds a substantial market share due to the growing demand of dental implant treatment (*Grand View Research, 2018,* Figs 1 and 2). The 2009 and 2010 National Health and Nutrition Examination Survey conducted by investigators at the Center for Disease Control and Prevention (CDC) pointed out that among adults in the USA, 8.7% have mild periodontitis, 30% have moderate periodontitis, and 8.5% have severe periodontitis [2]. Sixty-four percent of the population 65 years or older have periodontitis [2], indicating a large number of people in the USA are potential implant patients. With the fast increase in dental implant demand of $6.82 billion globally in 2024 as estimated upon data in *Grand View Research, 2018*, we are facing substantial challenges increase in peri-implantitis which is a challenge to long term survival of implants. A recent investigation indicates that the prevalence of peri-implantitis approximates 10% of implants and 20% of patients 5–10 years after implant placement [3]. Though investigators have revealed that the etiology of the peri-implant infection is similar to periodontitis [4–7], clinically there is no effective treatment. Implantation in patients with severe systemic conditions, i.e., severe diabetes, lupus, osteoporosis, organ transplant, and cancer with intensive radiotherapy and/or chemotherapy is another challenge to implant dentistry for lack of scientific research data. Animal models are crucial to help investigators reveal the mechanisms underlying these disorders. Variant animal implant models have been reported including mice, rats, rabbits, guinea pigs, dogs, sheep, goats, and nonhuman primates [8]. Genetically, both mice and rats are more than 90% similarities to human beings which are as high as all the other animals used to be implant animal models except for nonhuman primates. Biologically and economically, rats are the best animal models. In dental implant research, mice could not provide clinical compatible and macro-level measurable data for implant osseointegration and peri-implantitis in oral cavity due to lacking enough cancellous bone to support an implant more than 1 mm in length.

To create a clinically compatible rat model for implantation, we have done a comprehensive literature review of rat dental implant models. Implants placed out of oral cavity such as the femur [9–11] and the tibia [12, 13], or not on the ridge of alveolar bone, i.e., the ramus of mandibular [14] is not considered in the present experiment because these implantations are not clinically comparable. Freire and coworkers presented their work [15]. Briefly, the authors placed titanium tenting screw at maxillary medial suture and maxillary diastema mesial to the first molar. Promising results were obtained. An average 50.5% peri-implant osseointegration was observed in the implants placed at maxillary diastema of normal control which are significantly (*P* < 0.05) higher than peri-implant osseointegration (29.6%) in rats with experimental induced peri-implantitis [15]. Upon the reported 50.5% peri-implant osseointegration in normal control implants [15], we arrived at our hypothesis that implantation at maxillary diastema in rats could provide a high level osseointegration that consequently may allow to have a ligature induced peri-implantitis. Ligature induction is a commonly used method in induction of periodontitis in murine models [16, 17]. Ligature induced peri-implantitis will be more comparable than that of the pre-coating method reported by Freire and coworkers [15] due to the former is to induce inflammation after osseointegration of implant, but the latter is using a bacterial contaminated to induce inflammation at the time of implantation that will not be the case in regular clinical practice though as an experimental model, it still has its value to provide information of inflammation around an implant. Therefore, we designed our procedure to place implant at maxillary diastema and then induce peri-implantitis after its osseointegration (Fig. 1).

## Materials and methods

### Implant

Titanium bone screw with machined surface 1.2 × 4.5 mm (screw head 1.5 mm, fixture 3 mm) was purchased from ACS Surgical Supply (Brockton, MA) that was used as implant by Freire and coworkers [15].

### Animals

Six Sprague-Dawley, male, 400–450 g in which the body weight is based on reported article [15]. To achieve a stable implantation, the thickness of alveolar bone is crucial. Therefore, body weight is crucial for success of implantation. Animals were maintained and experiments were performed according to a protocol that was approved by the Rutgers Institutional Animal Care and Use Committee (IACUC).

### Designed experiment procedure

The time course is illustrated at Fig. 1. Rats were randomly located into 3 groups. Group 1 is for the baseline of 7 weeks after implantation, group 2 is for ligature induced peri-implantitis, and group 3 receives sham ligature to be the control of peri-implantitis 2 weeks after the procedure of ligature (Fig. 1a).

### Anesthesia

Anesthesia is achieved by intraperitoneal injection of ketamine HCl/xylazine solution in dose of 80 mg/kg/ketamine and 5 mg/kg/xylazine. During the anesthesia, breath and blood circulation were intensively monitored by observing chest and stomach movement, and color of the tip of nose and tongue. Animals were not sent back to the animal facility until the animals were completely awakened.

### Surgery and ligature design

Implantation is performed under anesthesia. When well anesthetized, the rat is laid in supine position. Jaws are kept open by pulling jaws up with silk suture loops tightened on upper and lower incisors. After cleaning the oral cavity with 0.12% chlorhexidine, a 0.5 mm × 0.5 mm niche in the alveolar ridge is made transmurally with a ball-shaped carbide dental bur with a diameter of 0.5 mm in a low speed to avoid overheating. Then, the implant is placed manually with an equipped screwdriver till no movement can be done. Each rat receives implantation of 1.2 mm × 4.5 mm titanium implants on the maxillary alveolar ridge in each side of the natural diastema (total of two implants per rat). Peri-implantitis is induced with ligature of silk suture at cervical part of implant that will lead to local inflammation and alveolar bone loss to mimic the clinical peri-implantitis. This procedure is modified from procedure to induce periodontitis [16, 17].

1. Baseline of osseointegration and induction of periodontitis

- *Seven weeks after the implantation*
  -Group (1): 2 rats will be sacrificed and osteointegration and inflammatory markers will be examined.
  -Group (2): 2 rats will receive suture ligature at the cervical part of each implant.
  -Group (3): 2 rats will receive no ligature as a control.

All the procedures will be performed when rats are under anesthesia.

(2) Identify induced peri-implantitis

- *Nine weeks after the implantation or 2 weeks after ligature procedure*

-Group 2 and group 3 will be sacrificed and osteointegration and inflammatory markers will be examined.

(3) Examination of implantation, osteointegration, and inflammatory markers:

- X-ray: periapical X-ray to examine the implantation of implants.
- Microcomputed tomography (Micro-CT)
  -The rat’s skull will be scanned with Micro-CT after sacrificed. (The dissected tissues will be taken to Rutgers Piscataway campus for Micro-CT examination as per IACUC policies.)

### Statistical analysis

Implant success and failure rate is analyzed by using Clopper-Pearson’s exact method at 95% confidence interval.

## Results

### Implantation

Under anesthesia, two implants were placed in maxillary diastemata in each rat (Fig. 2a). After implantation before the animal awakened, an X-ray was taken extraorally with a digital perioapical digital film. The X-ray indicates two implants were successfully placed in maxillary diastemata in one rat (Fig. 2b).

### Body weight change during the experiment

When rats arrived at animal facility, animals were stabilized for synchronizing. At day 50, rats were undergone implantation. One week after the implantation (day 57), a slight body weight loss of 2 g was observed. The body weight increase trend is recovered in 2 weeks after the implantation. As compared to the body weight continuing increase before the implantation, it indicates the procedure of implantation will have a slight effect on systemic condition (Table 1 and Fig. 1b).

**Table 1:**
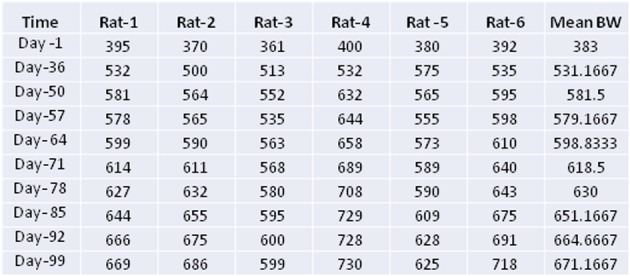
Rat body weight during the experiment

### Implant survival

Seven weeks after implantation, day 99 of the experiment, the rats were examined under anesthesia. Only one implant was survived and in a sounding condition without movement and sign of inflammation. X-ray indicates implant is located in maxillary diastema and surrounded by bone-like tissue (Fig. 2c). Implant success and failure rate is analyzed by using Clopper-Pearson’s exact method at 95% confidence interval. Our experiment data indicate that the true success rate of implantation in maxillary natural diastema in rat is less than 38.4% at a confident level of 95%. Since there is not enough data to construct a baseline of osseointegration with statistical significance (Fig. 1c), we ended the experiment at this time point and obtained samples for analysis.

### Implant location and osseointegration

**Fig. 3:**
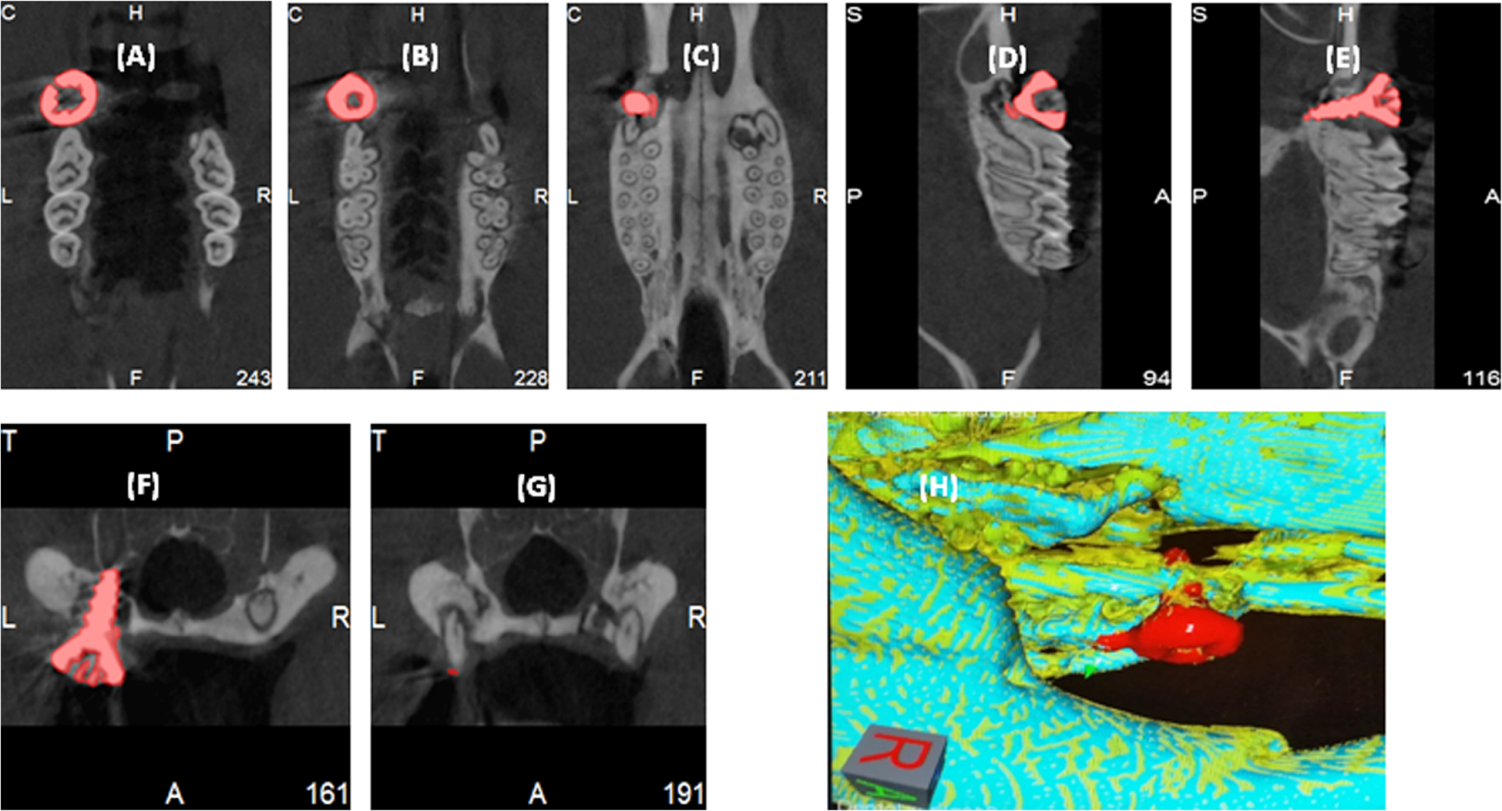
**a–c** Micro-CT frontal planes indicate the location of implant. **d**, **e** Micro-CT sagittal plane. **f**, **g** Micro-CT transverse plane. **h** Micro-CT 3D constructed image indicates osseointegration around the implant fixture

Micro-CT was used to examine the location and osseointegration of the implant, indicating the implant is located in the maxillary diastema without dislocation (Fig. 3a–g). Micro-CT 3D constructed image indicates osseointegration around the implant fixture (Fig. 3 h).

### A potential site for implantation

**Fig. 4:**
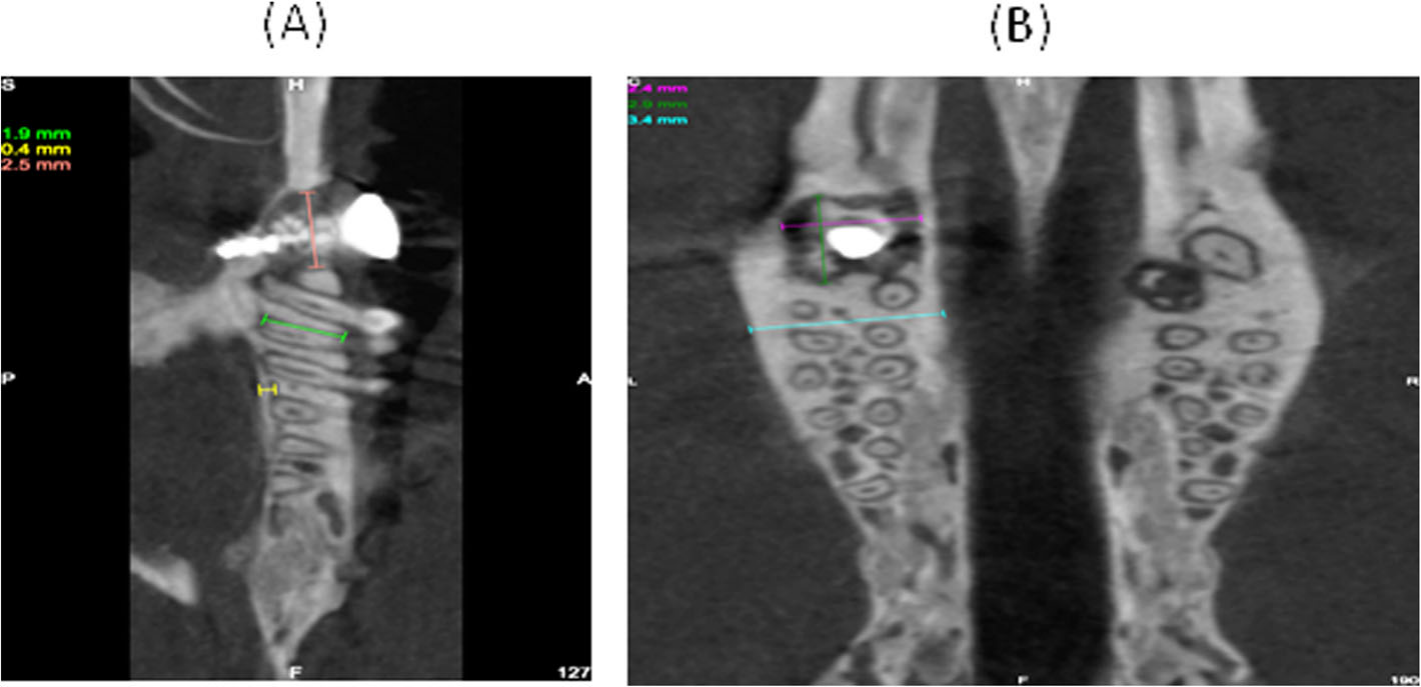
Micro-CT images indicate maxillary fist molar site will be able to provide a substantial cancellous bone to support a dental implant. **a** Sagittal plane. **b** Frontal plane

Micro-CT images indicate maxillary fist molar site will be able to provide substantial cancellous bone to support a dental implant (Fig. 4).

## Discussion

To explore the effective treatment for patients with severe systemic diseases and peri-implantitis, animal models are the most crucial subjects to help investigators to reveal the mechanisms underlying these disorders. Murine models both mice and rats are the most commonly used animal models in research because of their lower cost, biological relevance to human being, and available of genetic mutated strains. However, because of the body size of rat and mouse, it is difficult to have appropriate site in oral cavity to place implant as lager animals such as dog [18–21], sheep [22, 23], mini pig [24, 25], and non-human primate [26, 27]. To a successful implant, its fixture should be surrounded with cortical bone at cervical part and covered by cancellous bone at rest part of the fixture [28, 29]. Some investigators placed implant out of oral cavity such as the femur [9] and the tibia [12], or not on the ridge of the alveolar bone, i.e., the ramus of mandibular [14] to achieve osseointegration which are able to have samples for osseointegration; however, these models are not closely relevant to clinical implant placement. Implantation at diastema has been reported in mice [30]. However, the pictures showed in the articles, large portion of the implant (0.6 mm × 1.5 mm) are clear projected into the maxillary sinus though some peri-implant bone was observed by the authors. Clinically, it is not a normal practice to let large portion of implant fixture project into maxillary sinus. As the first of the article to place implant at maxillary diastema of rats, Freire and coworker provide informative research results [15]. They not only placed implant at maxillary diastema, but also placed implant at the hard palate midline area in the molar region. Anatomically, hard palate midline is a suture without cancellous bone to support the implant to form osseointegration so that it will not be a suitable place for implantation research.

Upon a comprehensive literature search, there are articles reported to place implants at maxillary first molar area. However, for variant reasons, these models are not clinical comparable. First, animal sizes are too small to have enough bone to support implant, i.e., Koutouzis et al. reported their experiment on rat model [31]. They placed diameter 1.5 mm × 2 mm length implants in approximately 9-week-old male Wistar rats in which maxillary first molars were extracted at week 5. It is just showed by figures in their article that the interradicular bone at the maxillary first molar obviously less than 2 mm, and the 2-mm length implant is projected into the sinus. And even in sham control rat (as showed in the article Fig. 5e, f), almost half of the implant was exposed in oral cavity without bone support, indicating body size of the rat is a crucial issue to take in consideration. Second, implant placement in clinically irrelevant position. Du et al. presented their research about place implant in 3-month female Sprague-Dawley rats [32]. Body weight is between 245 g and 279 g. They extracted the maxillary first molar and placed implant at mesial dental root socket. However, immediate implant placement at maxillary molar roots sockets in clinic should avoid for easily penetrating the floor of maxillary sinus and even dislocating the implant into the sinus due to the maxillary first, and second molar roots are close to the floor of sinus, particularly, in pneumatized sinus, so that interradicular bone in socket is the option to place implant. Therefore, the model reported by Du et al. is not clinically comparable. Third, bone of extract sockets has not appropriately formed. Inouye et al. [33] and Lin et al. [34] reported their experiments. They extracted the maxillary first molar from rats and placed implant at the socket area 4 weeks after the extraction. Histologically, bone formation starts at 4 weeks and complete around 24 weeks after the dental extraction; therefore, the socket has only soft tissue and immature bone at 4 weeks post extraction [35]. Clinically delayed implant placement must be in 12 weeks or more post extraction to wait for completion of alveolar bone formation [35, 36]. Thus, this model is not clinically relevant. Consequently, the maxillary first molar socket as an implantation site remains to be improved.

The current investigation followed the protocol depicted in the article by Freire et al. [15] and tried to modify the procedure of induction of peri-implantitis. Freire and coworkers were precoating the implant with bacteria to induce the inflammation. Our design is to induce the peri-implantitis after ossointegration of the implant. However, the implant survival rate is one per 12 of implant or one per six of rats and statistical analysis revealed that the implant true success rate is less than 38.4% at a confident level of 95% by using Clopper-Pearson’s exact method at 95% confidence interval. Therefore, we stopped the experimental protocol at 7 weeks after implantation and obtained samples for analysis. Osseointegration was clearly indicated by periapical X-ray and micro-CT. Meanwhile, micro-CT indicates the maxillary fist molar area could provide substantial cancellous bone to support an implant, indicating to be able to form osseointegration. Subsequently, the successful implant will allow to induce peri-implantitis on the base of osseointegration. The present research revealed maxillary natural diastema does not have substantial cancellous bone under the cortical bone. So even the implant achieves certain amount of osseointegration, it may not be able to further induce clinically relevant peri-implantitis. As a result, this report raises a question to dental implant model study about how to identify a clinical comparable animal model?

## Conclusion

According to the results of the present study, the authors have concluded the following outcomes:

- Current study indicates that maxillary nature diastema could be a site to place implant, but it has a low successful rate. Data indicate that the true success rate of implantation in maxillary natural diastema in rat is less than 38.4% at a confident level of 95%. Therefore, it is not an appropriate site for dental implant experiment. Moreover, it may be able to form certain osseointegration, but it could not provide enough cancellous bone to support an implant and further allow to induction of peri-implantitis on this implant. Further experiments are required to improve the successful rate of implant placement at maxillary nature diastema.
- Upon the intensively monitored body weight change during the experiment, rat body weight did not show abrupt changes after implantation though a slight decrease body weight at 2 g was observed 1 week after implantation, indicating the implantation surgery would not cause large interference in systemic conditions.
- Current investigation revealed a potential site that is maxillary 1st molar socket. After extraction of the maxillary 1st molar, the socket can maximally provide cancellous bone to support an implant 2 mm × 3 mm. Further experiment is needed to illustrate the details.

## Data Availability

N/A
